# Electromagnetic Fields, Genomic Instability and Cancer: A Systems Biological View

**DOI:** 10.3390/genes10060479

**Published:** 2019-06-25

**Authors:** Jonne Naarala, Mikko Kolehmainen, Jukka Juutilainen

**Affiliations:** Department of Environmental and Biological Sciences, University of Eastern Finland, FI-70210 Kuopio, Finland; mikko.kolehmainen@uef.fi (M.K.); jukka.juutilainen@uef.fi (J.J.)

**Keywords:** genomic instability, carcinogenesis, electromagnetic fields, systems biology, dynamical systems theory, attractor, state space

## Abstract

This review discusses the use of systems biology in understanding the biological effects of electromagnetic fields, with particular focus on induction of genomic instability and cancer. We introduce basic concepts of the dynamical systems theory such as the state space and attractors and the use of these concepts in understanding the behavior of complex biological systems. We then discuss genomic instability in the framework of the dynamical systems theory, and describe the hypothesis that environmentally induced genomic instability corresponds to abnormal attractor states; large enough environmental perturbations can force the biological system to leave normal evolutionarily optimized attractors (corresponding to normal cell phenotypes) and migrate to less stable variant attractors. We discuss experimental approaches that can be coupled with theoretical systems biology such as testable predictions, derived from the theory and experimental methods, that can be used for measuring the state of the complex biological system. We also review potentially informative studies and make recommendations for further studies.

## 1. Introduction

Health and disease are states of the human body, i.e., a biological system. It might therefore seem obvious that systems biology is needed in understanding environmental health effects. However, the term “systems biology” has been widely used only fairly recently for various approaches focusing on complex biological systems. It started to emerge as a distinct field of science with the advancement of high throughput omics technologies and with the improving capability of constructing computational models of complicated systems.

Although the approaches and methods used in systems biology are numerous, two (not mutually exclusive) main approaches can be distinguished. O’Malley and Dupré [1] define scientists using the first approach as “pragmatic systems biologists” and users of the second approach as “systems-theoretic biologists”. Huang [2] labeled these two categories as “localists” and “globalists”. Today, the majority of systems biologists fall in the former category, and for them systems biology simply involves the study of large-scale molecular interactions such as gene networks through the integration of multilevel data and models. The problem with this approach is that it is essentially reductionist and hinders research on system-level phenomena [3]. The latter approach is based on the general theory of complex systems. In this approach, a system is seen as a whole that is more than the sum of its parts: A complex system has properties, such as self-organization and emergence, that could not have been predicted based on analysis of the lower-level components.

Biological systems exist at different levels of organization such as cells, organs, and organisms. However, although many traditional areas of biology (e.g., neurology) deal with system-level biological functions, they are not called “systems biology”. Today’s systems biology approaches mostly center on deciphering cell-level phenomena, and cellular systems will also be the main focus of the discussion in this paper. However, as pointed out by Baverstock and Nikjoo [4], it should be recognized that each biological system has an environment with which it interacts, and at least part of this environment is a higher level system (cells are surrounded by tissues, tissues by organisms, and organisms by ecosystems). 

In general, much of present systems biology focuses on only modeling of biological systems. However, the ultimate goal should be interaction of theory and experimental science [3]. Even though mathematical modeling is central in understanding complex systems, it should be coupled to experimental in vitro and in vivo models in a two-way interaction: Models help to understand full implications of experimental findings, and they can generate predictions that can be tested in biological experiments.

This review discusses systems biology of electromagnetic fields, covering frequencies from extremely low frequencies (ELF; <300 Hz) to radiofrequencies (RF; up to 300 GHz). While several articles have applied various systems biology approaches to the biological effects of ionizing radiation [4,5,6,7,8,9] and chemicals [10,11], systems biological approaches to biological effects of electromagnetic fields have been discussed, to the best of our knowledge, in only one book [12]. We focus particularly on induced genomic instability and its role in cancer as cancer is a major issue in the discussion of the health effects of electromagnetic fields (and in general in environmental health). Moreover, cancer is obviously a phenomenon that emerges at a system level in a living organism, and there has been great interest in using systems biology to gain insights into carcinogenesis [6,7,9,13,14,15,16]. We will discuss the use of systems biology (the systems-theoretic approach) in understanding genomic instability and carcinogenesis, its application to theoretical and experimental studies on the effects of electromagnetic fields, and future directions of the systems biology of electromagnetic fields.

## 2. Dynamical Systems Theory in Biology: Basic Concepts

Biological entities like cells are very complex systems consisting of multiple feedback loops that are either positive or negative. The behavior of such complex systems includes self-organization (generation of some form of order from the interactions of the parts of the system) and emergence (appearance of system properties that its isolated parts do not have, generally not predictable from the lower level properties) [2,4]. We will briefly introduce the discipline of dynamical systems and its applications to biology.

A central concept of dynamical (i.e., time-dependent) systems is that of *state space*. The *state* itself can be defined as a complete description of a physical system i.e., everything that can be possibly known about it. The state space of a dynamical system is then the set of all possible states of the system. Each coordinate of the state space is a state variable, and the values of all state variables completely describe the state of the system. Consequently, each point in the state space corresponds to a different state of the system. 

The time evolution of a dynamical system is modeled using continuous or discontinuous description (differential or difference equations) of the form:

d*x*/d*t* = F(*x*,*u*,*t*)  (continuous), (1)

*x_n_*_+1_ = f(*x_n_*_,*u*_)  (discontinuous) (2)

where variables *x* span a state space, *t* is time, *n* is index of discrete time, and *u* refers to parameters of the functions F and f. The trajectories (time developments) in the state space advance toward limit sets called attractors. In principle four types of attractors can exist [12]:

- Fixed point: static attractor;
- Limit cycle: periodic attractor;
- Torus: quasiperiodic attractor;
- Chaos: strange attractor.

The systems modelled usually have an inner structure where different parts of the process influence each other [17]. These complex interactions usually include lags, i.e. the changes in one part of the system affect some other part of it with a delay. This in turn is reflected in the corresponding measured time series as lagged values. Complexity in many natural phenomena arises from massive interactions among different parts of a non-linear dynamical system [17]. If the system is additionally sensitive to initial conditions, it can express *chaotic* behaviour. In such a system, nearby states of the system are separated rapidly. This leads to diminished ability to forecast the future evolution of the system, thus effectively preventing the accomplishment of the short time goals of time-series forecasting. Chaos and random noise are expressed similarly in a time series, making it difficult to distinguish the former from the latter. More compactly, chaos is defined to be [18] “*…aperiodic, long-term behaviour of a bounded, deterministic system that exhibits sensitive dependence on initial conditions.”*

A simple example of the deterministic chaos phenomenon is the so-called logistic map:

(3) $$x_{t+1}(\mu )=\mu x_{t}(1-x_{t})$$

where *µ* is a control parameter determining the amount of “energy” affecting the system. This model has been used to model simple predator-prey systems (like rabbits and wolves). Even though the equation appears to be quite simple, it can demonstrate complex phenomena through so called bifurcation route, which is one explanation for the appearance of chaotic behaviour in deterministic systems.

**Figure 1:**
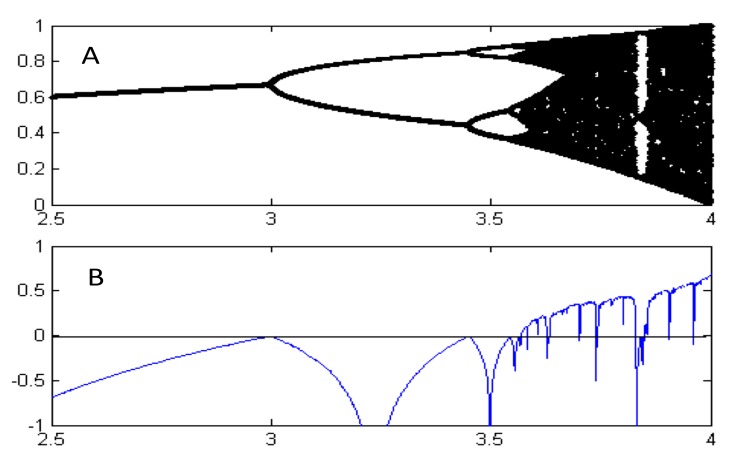
**A**. Bifurcation diagram showing the set of values of the logistic function visited asymptotically at different values of the bifurcation parameter *µ*. **B**. The corresponding Lyapunov exponent, which is often used for quantifying the sensitivity of a system to initial conditions [12]. A positive sign of the exponent signifies chaos and its value measures its quantity. Note that the chaotic regimes in A correspond to positive Lyapunov exponent values in B.

**Figure 2:**
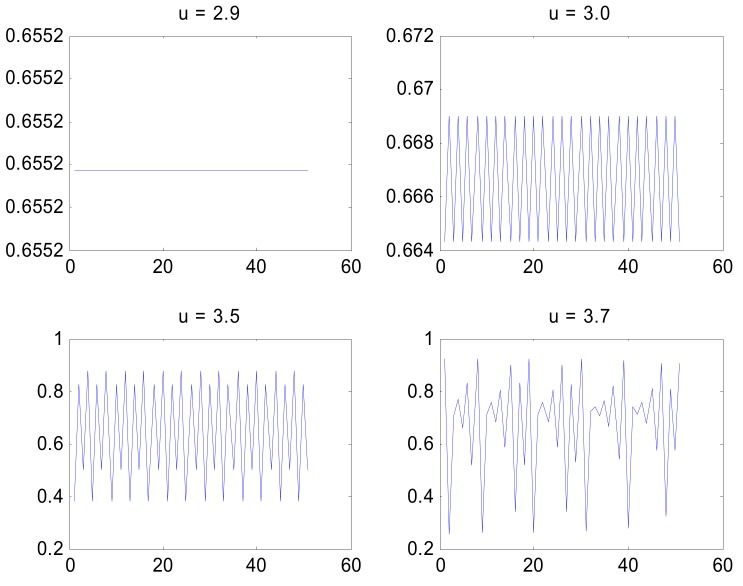
The behaviour of the logistic map with different values of the control parameter *µ*. The map has been iterated for 10000 times and the last 50 values of the time series are shown here.

The first bifurcation (doubling of the number of attractor points) appears when *µ* reaches the value of 3.0 and the next at approximately 3.45. The bifurcations appear after that with an accelerating speed and the crucial value for chaos *µ*_∞_ is reached with an approximate value 3.57, as shown in the bifurcation diagram in Figure 1. For analytical derivation for these values see Rasband [19], for example. The time-dependent behavior of a fixed point (*µ* = 2.9), periodic (*µ* = 3.0 and *µ* = 3.5), and strange attractor (*µ* = 3.7) have been illustrated in Figure 2 where the tail of a long time series has been plotted to characterize the behavior of a system trapped in one of its attractors.

**Figure 3:**
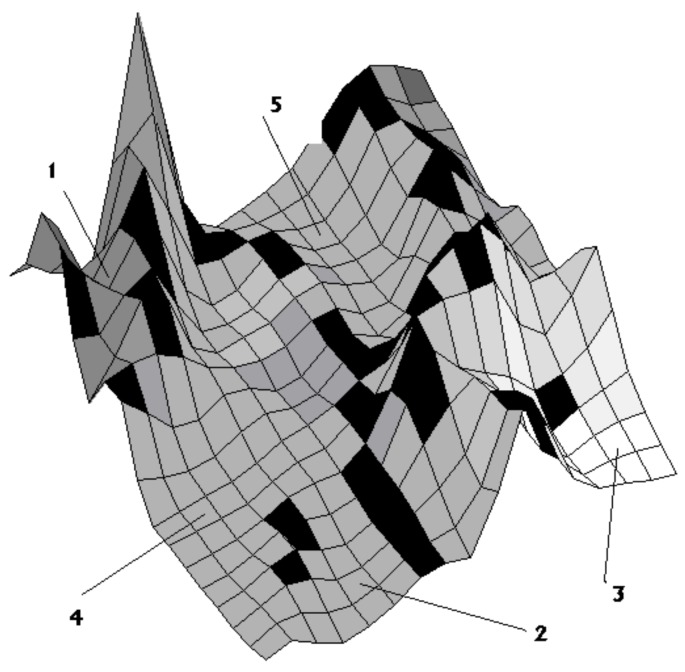
Visualization of a state space landscape with five basins (1–5; areas in the state space) of attraction.

In order to visualize the attractor structure of a multidimensional biological system (in contrast to the one-dimensional logistic map), several authors (e.g., [2,20,21,22]) have suggested the use of state space landscapes. Such a landscape can be illustrated with a three-dimensional picture where the *x*- and *y*-axis correspond to the state space variables (the multiple variables of a true biological system represented by just two variables) and the *z*-axis to a “potential” parameter. This kind of landscape is shown in Figure 3 where there are five “basins of attraction” (sets of points from which the system spontaneously moves into the attractor; in Figure 3 numbers 1–5) around an attractor (lowest point of *z*) in each basin.

## 3. Genomic Instability

There is increasing evidence that induced genomic instability (IGI) plays a role in environmentally induced cancer. IGI is a concept describing the delayed damage which can be observed in many cell generations after exposure in the non-exposed progeny of exposed cells as increased mutation frequency, apoptosis, chromosomal aberrations, micronuclei, and other damage [23,24,25]. Originally IGI was found in cells exposed to ionizing radiation, but several other chemical and physical agents have been reported to induce genomic instability [26,27,28,29,30,31,32]. In addition to cell cultures, IGI [33] has been observed in animals and even in humans [34,35,36,37,38,39,40,41,42]. 

A phenomenon closely related to IGI is genomic instability in cancer cells, which typically display high number of mutations [33,43,44]. Although cancer-associated genomic instability is a characteristic of the late stages of cancer and therefore not necessarily identical to the IGI encountered rather soon after exposure environmental stressors, it is rational to postulate that these two phenomena are related. 

IGI is initiated and transmitted epigenetically [45], which refers to heritable changes in the gene expression or in the phenotype that are not attributable to changes in the DNA sequence [46]). Epigenetic mechanisms are considered to include, e.g., DNA methylation signature, histone modifications, ubiquitination, and sumoylation processes, and non-coding RNAs [47], but other mechanisms for transmission of epigenetic information may exist. 

As the process of carcinogenesis requires accumulation of multiple genetic changes, IGI is obviously important in the development of cancer [48]. However, its health implications may be much wider as accumulation of DNA alterations and increased level of reactive oxygen species— another characteristic of IGI [37]—seem to play key roles in the development of other chronic diseases such as cardiovascular and neurodegenerative diseases [49]. Although the maintenance of genome stability is crucial for the well-being of higher organisms, IGI has also been seen as a resource for evolution and a mechanism of adaptation [50].

## 4. Dynamical Systems View of Genomic Instability and Cancer

**Figure 4:**
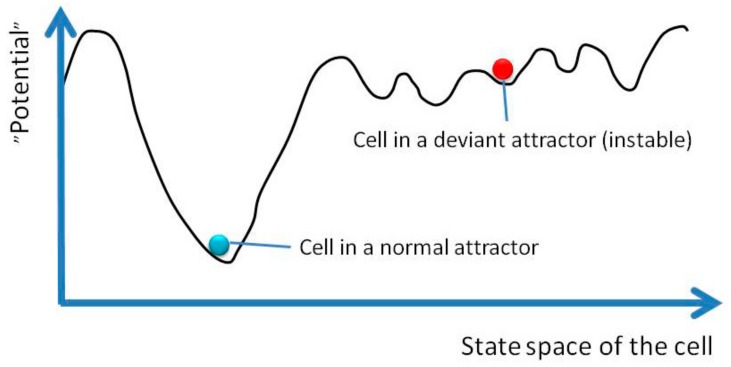
Schematic representation of cells in normal and variant attractors in one-dimensional projection of the state space.

Systems biological approaches to carcinogenesis and genomic instability have been proposed by Baverstock [23,51] and Huang [13,20]. Both proposals include the idea that cancer cells represent abnormal attractor states. We start from the view that each cell phenotype corresponds to an attractor, i.e., a specific state with characteristic gene expression pattern, and differentiation corresponds to movement in the landscape (state space), which is determined by the genome. In normal development towards differentiated cell types, the cells move along landscape-determined trajectories through transient states to a stable attractor representing a mature cell type. However, the complex multidimensional state space (landscape) most likely contains, in addition to the normal attractors, numerous attractors (valleys in the potential landscape) that are not normally used by the cells (Figure 4). These unused attractors, termed “variant attractors” by Baverstock, have not been evolutionarily optimized and are therefore less stable than normal attractors: The basins of attraction (valleys) are shallow compared to those of the evolutionarily conditioned attractors [51]. As a consequence, the cells in a variant attractor are prone to environmental perturbation and relatively easily migrate into other variant attractors. This loss of stability of the system is hypothesized to correspond to the genomic instability seen in cancer cells (Huang) and to genomic instability induced by radiation and other environmental agents (Baverstock). Moving from the evolutionarily conserved normal attractors to the normally unused region of the state space involving variant attractors can happen due to mutations that change the epigenetic landscape (=genome); reducing the depth of an attractor or flattening out of the “hills” that surround it will make it easier for a cell to “climb” from the attractor basin to the region occupied by variant attractors (Figure 4). This type of instability corresponds to the mutation-dependent genomic instability seen in advanced cancer. However, also a non-genetic route into a variant attractor has been described [51]—the cellular system may be forced to leave the normal attractor (jump over the hills in the quasi-potential landscape) due to an environmental perturbation, such as a large enough dose of ionizing radiation. Once trapped on the other side of the “hills” away from the normal attractor in the state space occupied by variant attractors, the cells would not be likely to return to the normal attractor. This second type of instability corresponds to genomic instability induced by environmental agents [23,24,25], which has been shown to be primarily an epigenetic phenomenon, in which delayed genetic changes are a consequence rather than a prerequisite of the instability of the system (see Section 3). 

## 5. Experimental Approaches

In this section we will discuss experimental approaches that might help to understand system-level responses to environmental agents such as electromagnetic fields. Although the majority of systems biology literature is theoretical, experimental studies are essential for the development of the field. Theory is useless if it cannot be tested empirically. 

### 5.1. Predictions That Can Be Experimentally Tested

This paper discusses the systems biological approach only at a qualitative level, so no quantitative predictions can be presented. However, several testable qualitative predictions can be derived. 

We start with the hypothesis that induction of genomic instability corresponds to transition into the basin of a variant attractor state. The system is then instable because the barriers between alternative variant attractors are shallow, and the system keeps drifting form one attractor to another. As each attractor corresponds to a specific pattern of gene expression, induced genomic instability should be associated with increased variation in gene expression. In the case of ionizing radiation, there is empirical evidence that fits with the predicted increase in the heterogeneity of gene expression. Fält et al. [52] found that the gene expression pattern in irradiated human lymphocyte clones cultured for multiple cell generations after irradiation was more diverse than in non-irradiated clones. The gene expression profile also changed strongly over time, with very few genes consistently up- or downregulated at all time points studied. The increased heterogeneity (as predicted by our hypothesis) thus seems to be observable both between cell clones and between time points after irradiation. As the gene expression measurements by Fält et al. [52] were done many cell generations after irradiation, the results most likely reflect radiation-induced genomic instability rather than direct responses to radiation-induced damage. However, the study did not include measurement of any other endpoints than gene expression to confirm radiation-induced genomic instability by independent methods. Later, studies showed increased diversity of gene expression in the progeny of irradiated *Caenorhabditis elegans* nematodes [53] and in the progeny of irradiated murine fibroblasts [54]. These studies included also measurements—delayed mutations in *C. elegans* and delayed induction of micronuclei in cultured fibroblasts—that confirmed the presence of induced genomic instability in these experimental models. The divergent pattern of changes in IGI may not be limited to changes in gene expression. Very heterogeneous abnormalities developed in the karyotypes of irradiated one-cell-derived cell clones that expressed genomic instability [55,56]. 

Another prediction that follows from the variant attractor hypothesis is that induced genomic instability should be associated with unspecific changes in gene expression. As described above, we assume that there are a high number of variant attractors in the state space of the cellular system. A cell that becomes unstable jumps into a variant attractor and thereafter between variant attractors (=different gene expression patterns) essentially in a random fashion, and there is no reason to assume that we would see a gene expression pattern that is specific to genomic instability. Gene expression data measured in cells and nematodes exposed to ionizing radiation fit with this prediction; while typical gene expression changes associated with DNA damage response, cell cycle control, and apoptosis are observed shortly after irradiation [54], measurements performed at delayed time points (corresponding to genomic instability persisting in the progeny of irradiated cells) show only unspecific changes with no clear clustering into certain categories of genes [52,53,54].

### 5.2. How to Measure the State of the System? 

We have described above the biological system in terms of attractors within a multidimensional state space that emerges as a result of the complex regulatory network of the cell. We have also derived some predictions that can be tested empirically. Empirical testing requires methods for making observations, so how can we measure the state of a biological system? As the normal attractors correspond to differentiated cell types, observing cell phenotypes (morphology, biochemical markers) can be used in certain areas of biology, such as developmental biology, to determine the state of the biological system [16,57]. However, the variant attractors of “diseased” cells do not correspond to any well characterized differentiated cell phenotype, so measuring attractors as cell phenotypes is not straightforward in this case. 

As all levels of the biological system from genes to metabolites are interlinked, any omics approaches (e.g., transcriptomics, proteomics, metabolomics) should be useful for detecting changes in the state of a biological system. Omics methods include measurements of a very high number of gene products or metabolites and can therefore capture the complexity of the multidimensional state space representing a biological system. However, as we will show below, the omics methods may not be the only (and possibly not the optimal) approaches for observing the state of the multidimensional biological system.

The attractors of biological systems are typically described as static states such as gene expression patterns [13]. In such approaches, a specific state of the system (e.g., attractor) is thought to be measurable as a high level of certain gene products and/or low level of certain other gene products. This approach corresponds to modeling cellular states as fixed-point attractors, using the terminology of the dynamical systems theory. However, there is evidence that everything oscillates in true living biological systems [58,59,60,61]. In addition to the well-known circadian rhythm and the cell cycle, there are ultradian rhythms, biological oscillations with periods down to milliseconds. The true biological attractors may therefore be periodic (cyclic or chaotic) attractors rather than point attractors. The oscillating nature of biological systems does not mean that the stationary omics approaches cannot be used for getting some information about the state of a system; in spite of the continuous, multi-frequency oscillations, it can be assumed that the *time-average* levels of gene products have specific patterns associated with attractor states. However, understanding the biological system as a complex dynamical oscillator opens new insights into measuring the system state and observing attractors. As the oscillations of the components of the biological network are correlated (for example, a peak in mRNA level of a gene could lead to a peak in the activity of an enzyme and to a corresponding change in the level of a metabolite), it is possible to observe the state of the system by real-time monitoring of just a few variables (see 3.3). Indeed, Roussel and Lloyd [62] have shown a chaotic metabolic attractor by measuring just dissolved oxygen, carbon dioxide, and hydrogen sulfide in yeast cells that showed oscillations with periods of 13 h, 36 min, and 4 min.

### 5.3. Review of Potentially Informative Studies 

In principle, any omics data from studies using high throughput technologies is potentially useful for a systems biology analysis. Several research groups have investigated transcriptomic, proteomic, or metabolomic changes following exposure to ELF or RF electromagnetic fields. Earlier studies have been reviewed [63,64], and additional studies have been published recently [65,66,67,68,69,70,71,72]. There are many limitations in individual studies such as short exposure times, limited range of exposure levels, and limited number of sampling times after exposure. Nevertheless, the studies together cover a wide range of exposure levels, exposure durations, and many different in vitro and in vivo experimental models. Most of these studies have found no effects of electromagnetic field exposure using conventional bioinformatics approaches. Many of the early studies reported a few exposure-related changes using high throughput technologies, but the number of positive findings was often below the expected frequency of false positives, and the studies did not include confirmation of the findings using independent methods such as RT-PCR. Overall, the studies conducted this far have not provided evidence of transcriptomic or proteomic changes that would be consistently observed in several studies. A possible explanation for this lack of consistency is that there are no effects from exposure to electromagnetic fields, and the reported positive findings are just random noise. Another possible explanation is the wide variation in experimental details (such as cell or animal models, frequencies, field intensities, exposure durations, sampling times post exposure) that may have resulted in varying responses in different studies. Moreover, responses of a (complex) biological system may not always involve significant changes in measures of the average expression levels of single genes or proteins. Instead, deregulation of a biological pathway may be observable as increased variance of expression levels, with no differences in the median expression levels. This has been demonstrated in comparisons of expression data from cancer patients and healthy controls [73], and increased variance might as well be caused by exposure to environmental agents, particularly if genomic instability is induced (see 6.1). 

The omics studies evaluating biological effects of electromagnetic fields have generally relied on standard bioinformatics analysis tools and have not attempted to use more advanced systems biological approaches. Parham et al. [74] examined linkages of ELF and RF electromagnetic fields to biological pathways and to classes of human disease (via their connections to biological pathways) using microarray data from several previous in vitro studies. The RF electromagnetic field datasets were not clearly linked to any disease classes but did show linkages to changes in several pathways such as apoptosis, cellular regulation, and cytoskeleton maintenance. The ELF datasets supported links to disease classes (cancer, chemical dependency, metabolic dysfunction, and neurological disorders) but links to pathways showed very little consistency. The links to disease classes were largely driven by a single study, precluding any firm conclusions. This study was limited by the inhomogenous study designs (with respect to, e.g., exposure characteristics and cell lines used) and small microarray data sets included.

Given the oscillating nature of biological systems (see 6.1), responses to external agents can also involve changes in the frequency of the oscillations, as shown in a study investigating transcript oscillations in yeast cells exposed to phenelzine [75]. Thus, studies on biological oscillations may be informative with respect to responses of biological systems to EMFs. As discussed above, omics approaches are not needed in this kind of studies as detecting a chance in a few oscillations (or maybe just one) may reveal changes in the cellular state consisting of multiple correlated oscillations. From this point of view, it is of interest whether EMFs can induce changes in circadian rhythms and cell cycle. Sensitivity of the circadian clock to EMF has been reported in *Drosophila* [76,77] and suggestive evidence of magnetic field effects on circadian rhythms has also been found in mice and cows [78,79,80]. Furthermore, EMF effects on the expression of circadian clock genes were reported in a human fibroblast cell line [81]. Other studies have reported changes in cell cycle distribution [82,83,84,85,86,87] and altered expression of genes and proteins involved in cell cycle regulation [84,85,86,87,88,89]. These studies did not include repeated measurements of any cellular parameters at short intervals and could therefore not evaluate possible changes in cellular attractor states such as those shown for some other exposures [60,75]. However, the circadian rhythm and cell cycle are linked to other biological oscillations, including ones with shorter (ultradian) time scales, such as oscillations in gene and protein expression, metabolite oscillations, and the redox cycle [59,90]. It therefore seems likely that valuable insights could be gained from studies investigating EMF effects on fast cellular oscillations.

Possible induction of genomic instability by EMFs is of particular interest, because IGI may be systems biological by its very nature—a change of the dynamic biological interaction network as a response to external stress [91]. Four studies have addressed induction of genomic instability in vitro in the progeny of cells exposed to ELF magnetic fields and all reported positive findings. In the first study, bleomycin-induced chromosomal instability was enhanced in human fibroblast cells by a 60-Hz, 800 µT magnetic field applied continuously for up to 240 h after the bleomycin treatment [92]. Mairs et al. [93] analyzed microsatellite sequences in human glioma cells exposed to 50 Hz, 1 mT magnetic fields for 12 h, either alone or combined with exposure to ionizing radiation before magnetic field treatment. One of the three mutation types that were evaluated was allelic imbalance. This type of genetic change is caused by allelic loss occurring in the progeny of the exposed cells during the post-irradiation exposure incubation, and therefore indicates IGI. The frequency of microsatellite mutations was increased 38 d after the treatments, and this increase was particularly pronounced for allelic imbalance. Two recent studies reported increased frequencies of micronuclei 8–30 days after exposure in the progeny of human neuroblastoma cells exposed to 50 Hz, 100 µT magnetic fields for 24 h, with or without combined treatment with menadione [94,95]. In a study using similar experimental approach, no genomic instability was induced in rat primary astrocytes exposed to GSM-modulated 972 MHz RF EMFs at specific absorption rates of 0.6 or 6 W/kg [96]. Induction of genomic instability by a third type of EMF, intermediate frequency (IF) magnetic field, was also tested using rat primary astrocytes. Exposure to a 7.5 kHz, 300 µT field for 24 h resulted in decreased level of micronuclei 36 days after the exposure, i.e., this treatment seemed to reduce genomic instability [97]. None of these studies on EMF effects involved measurements, such as omics or cellular oscillations, that would allow systems biological analyses. However, we believe these studies provide an excellent starting point for further studies; it will be highly interesting to test our predictions (see 6.1) and novel measurement methods (6.2) in experimental systems that have shown increased genomic instability in response to ELF magnetic fields or reduced genomic instability after exposure to IF magnetic fields.

## 6. Future Outlook

The ultimate goal in future systems biological studies should be interaction of theory and experimental science [3]. Even though mathematical modeling is central in understanding complex systems, it should be coupled to experimental in vitro and in vivo models in a two-way interaction; models help to understand full implications of experimental findings, and they can generate predictions that can be tested in biological experiments.

Any existing omics datasets on EMF effects might be utilized for testing for agreement with two of the systems biological predictions that we derived in Section 5.1: If genomic instability is induced, (1) heterogeneity of the data (mRNA expression, protein expression, metabolite levels) should increase in the exposed cells in comparison to control cells and (2) the changes observed should be unspecific. Note that the latter is not equal to showing no consistent changes; one should be able to show with sufficient confidence that there are statistically significant exposure-induced changes, but they should be different between, e.g., cell clones. Therefore, existing omics datasets may not include data suitable for testing the second prediction. 

Studying cellular oscillations as a measure of the state of the biological system (5.2) will require novel experiments that include repeated measurements with short intervals to allow detection of changes in periodic (cyclic, chaotic) attractor states. As measurement of a few variables is sufficient to follow the oscillatory cellular dynamics, methods such as live cell imaging may let us understand whether and how EMFs affect such dynamics, possibly resulting in changes in the state of the biological system. We recommend this as the most promising avenue for future experimental approaches in systems biology of EMFs. 

Although above we have mainly focused on genomic instability and cancer, we believe our final recommendation is relevant to many other biological responses as any biological effects relevant to human health (such as developmental effects or effects on the nervous system) are most likely associated with changes in the state of the biological system, and the methods outlined above may give insights into the fundamental interaction leading to biological effects of EMFs.
